# Cell wall water shields stomata against falling leaf airspace humidity

**DOI:** 10.1111/nph.70998

**Published:** 2026-02-08

**Authors:** Michael R. Blatt, Adrian Hills, Tracy Lawson, Julie Magill

**Affiliations:** ^1^ Laboratory of Plant Physiology and Biophysics, Bower Building University of Glasgow Glasgow G12 8QQ UK; ^2^ Department of Plant Biology, College of Liberal Arts and Sciences and Institute for Genomic Biology University of Illinois 1206 W Gregory Dr. Urbana IL 61801 USA; ^3^ MVLS Mechanical Workshop, Kelvin Building University of Glasgow Glasgow G12 8QQ UK

**Keywords:** cell wall water potential, guard cell, stomatal transpiration, substomatal cavity, vapour pressure deficit, *Vicia faba*

## Abstract

- Plants lose water by transpiration through stomatal pores. However, it remains a matter of debate whether relative humidity (RH) in the substomatal cavity may fall below saturation and guard cells experience strong differences water potential driven by RH in the cavity. We developed a gas exchange chamber to control RH and CO_2_ at the inner epidermal surface.
- *Vicia faba* L. stomata remained open with high stomatal conductance (*g*_s_), even when RH inside was reduced substantially below saturation.
- Concurrent measurements showed no resolvable decline in bulk cell wall water potential, even with 50 %RH inside, provided the wall space was hydrated. Only when the tissue was allowed to dry did the wall water potential fall below −2 MPa, the stomata close, and *g*_s_ collapse to values near zero. These findings concurred with OnGuard model predictions showing large decreases in RH in the leaf under water stress.
- The observations highlight a steady‐state flux from liquid in the cell wall to vapour in the substomatal cavity and across the stomatal pore; they implicate cell wall water in shielding the stomata against leaf airspace humidity; and they pose a challenge to consider the kinetics of evaporative flux behind stomatal transpiration.

## Introduction

Plant leaves lose water by transpiration through stomatal pores when the relative humidity (RH) outside the leaf falls below that of the airspace within the leaf. Water rising through the xylem to the leaf passes into the cell walls of the leaf mesophyll, epidermal and guard cells. It evaporates from the cell wall surfaces inside the leaf before diffusing through the stomatal pore to the atmosphere. In these circumstances, a standing gradient must arise between the water potential in the cell wall, Ψ_wall_, and the airspace within the leaf, extending through the substomatal cavity to the atmosphere. Nonetheless, it has remained a matter of debate whether the water potential, and hence RH, of the airspace of the substomatal cavity behind the stoma may fall substantially below saturation, especially at high vapour pressure differences (VPDs) between inside the leaf and the atmosphere.

Assuming that high VPDs give rise to unsaturation in the substomatal cavity, it may be seen to reduce the apparent stomatal conductance, *g*_s_, through a mechanism separable from that of stomatal movements (Jarvis & Slatyer, 1970). A fall in the water vapour content in the substomatal cavity would reduce the VPD across the stomatal pore itself, thereby reducing the driving force for transpiration through the stoma without a change in stomatal aperture. Livingston & Brown (1912) proposed ‘incipient drying’, a retreat in the margins of the wetted surface within the leaf under high evaporative pressure. More recent analysis of hydration profiles within the leaves of woody species supports this idea, indicating a retreat of the fully wetted surface that adds a ‘wall resistance’ component to transpiration when water supply to the transpiring leaf was reduced (Rockwell *et al*., 2014). Measurements of this added resistance led to early suggestions that RH behind the stomatal pore might reduce to values near 70% at high VPDs (Jarvis & Slatyer, 1970). Subsequent studies have either yielded evidence of RH reductions (Egorov & Karpushkin, 1988; Canny & Huang, 2006; Cernusak *et al*., 2018; Cernusak *et al*., 2019; Diao *et al*., 2024; Jain *et al*., 2025) or failed to support a substantial RH decline within the leaf airspace (Farquhar & Raschke, 1978; Holloway‐Phillips *et al*., 2019).

The idea that RH of the substomatal cavity might fall substantially below saturation is problematic for several reasons (Rockwell *et al*., 2022). Among these, gas exchange calculations of photosynthetic carbon assimilation rely on transpiration to quantify the conductive pathway for CO_2_ entry to the leaf. These calculations assume that the airspace within the leaf is at, or very close to, saturation with water vapour. Were the airspace behind the stomatal pore to fall far below 100% RH, then the calculations of *g*_s_ and of the partial pressure of CO_2_ (pCO_2_) within the leaf airspace, pC_*i*_, would underestimate true values and propagate similar errors in the calculated rates of carbon assimilation. Furthermore, as Buckley & Sack (2019) have pointed out, declines to 70–80 %RH at equilibrium within the inner airspace of the leaf equate to water potentials near −30 to −40 MPa, values far below a water potential of −3 MPa that is sufficient to plasmolyse virtually all plant cells in solution. Indeed, the root of much scepticism centres around the physiological status of the leaf tissues, especially of the guard cells, in the face of such steep gradients in water potential. Simply put, how could guard cells survive such extremes in water potential?

**Fig. 1:**
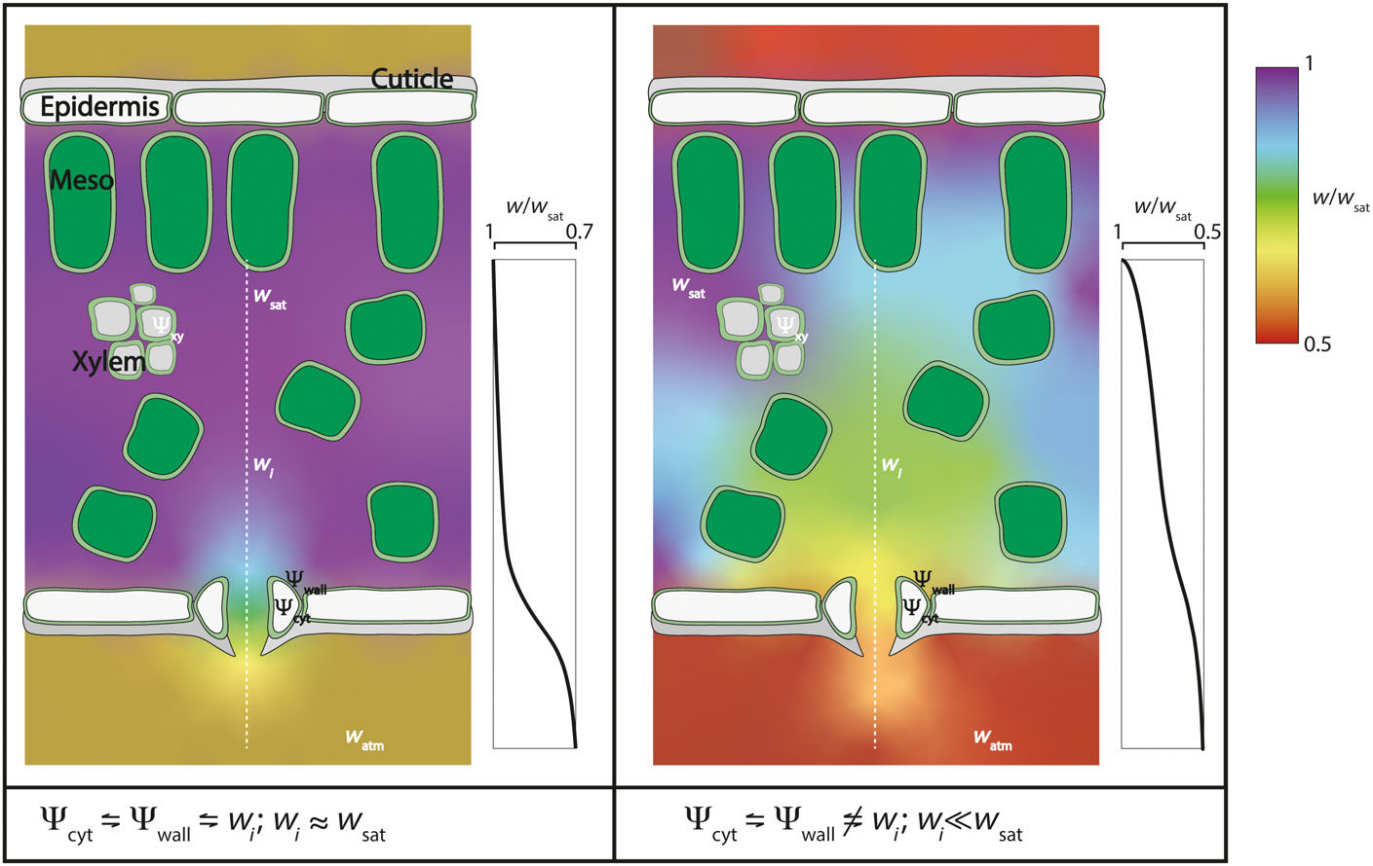
Spatial distribution of water vapour partial pressures between saturation and gradients and water potentials of the xylem guard cell and cell wall. Diagrams correspond to a water‐replete plant in higher atmospheric water vapour partial pressure (left, low Δ*w*) and a water‐stressed plant in low atmospheric water vapour partial pressure (right, high Δ*w*). Here, Δ*w* = *w*_sat_ − *w*_atm_, *w*_sat_, *w*_*i*_ and *w*_atm_ are the water vapour partial pressures at saturation, in the substomatal cavity, and in the atmosphere, respectively. The water potentials Ψ_xy_, Ψ_wall_ and Ψ_cyt_ correspond to the xylem, cell wall and guard cell, respectively. Relative humidity (=*w/w*_sat_) along the dotted line is plotted to the right of each diagram. When water‐replete with low Δ*w*, *w*_*i*_ ≈ *w*_sat_ and the relative humidity drops primarily across the stomatal pore; under water stress with high Δ*w*, relative humidity drops away from the evaporative surfaces with a substantial component to Δ*w* occurring across the substomatal cavity and a smaller fraction of Δ*w* across the stomatal pore.

Transpiration occurs when there is a concentration gradient in water vapour from the sites of evaporation within the leaf to the atmosphere. Such gradients imply that the air space within the leaf must fall below water vapour saturation (Fig. 1). A saturated water vapour partial pressure, *w*_sat_, may be found at the sites of evaporation within the leaf, but we can expect a gradient in humidity to arise across the substomatal cavity and through the stomatal pore in order to support the diffusive flux in water vapour. When the difference between *w*_sat_ and the water vapour content of the atmosphere, *w*_atm_, is small – for example, with Δ*w* < 6 mmol mol^−1^ – then it is reasonable to expect the bulk of this gradient to occur across the stomatal pore with little decline in water vapour content in the substomatal cavity, *w*_*i*_. In this case, the *w*_sat_ profile may be thought to form a standing gradient within the leaf with a ‘near‐saturation edge’ by the pore itself so that *w*_*i*_ *≈ w*_sat_ and the conductive pathways for water vapour and CO_2_ diffusion are approximately the same through the stomatal pore and the leaf airspace (Moss & Rawlins, 1963; Morison *et al*., 2007). Stomatal conductance to water vapour, *g*_s_, then provides a reasonable proxy for the stomatal conductance to CO_2_ when scaled by the difference in concentrations and gaseous mobilities.

When Δ*w* is large however, and especially when water flow to the leaf is restricted, a larger fraction of the standing gradient in water vapour content is likely to extend inwards into the inner leaf airspace. Whether this extension is described by a shift in the ‘saturation edge’ (Wong *et al*., 2022), in the margin of incipient drying (Livingston & Brown, 1912), or in the balance between liquid and gaseous water transport within the leaf (Rockwell *et al*., 2014), the standing gradient in water vapour content is likely to expose the guard cells to reduced *w*_*i*_ and RH (=*w*_*i*_/*w*_sat_) with *w*_*i*_ << *w*_sat_.

For many plants, the bulk water potential of a leaf, Ψ_leaf_, situates between −0.5 and −1.0 MPa. Ψ_leaf_ accounts for the whole leaf and incorporates a large component determined by the osmotic potential of solutes within the cells of the leaf, Ψ_cyt_. Were this water to equilibrate within the substomatal airspace, it would give *c*. 98–99 %RH. By the same token, equilibrated to 90 %RH would imply a value for Ψ_leaf_ of −14 MPa, more than sufficient to collapse every cell within the leaf (Edwards *et al*., 1976; Edwards & Meidner, 1979; Cosgrove, 1987; Meshcheryakov *et al*., 1992). These considerations raise a straightforward question: Do guard cells experience such strong differences in water potential across the plasma membrane driven by *w*_*i*_? Addressing this question *in situ* is not practicable. However, we can ask whether stomata in isolation operate when *w*_*i*_ in the leaf airspace and the water potential of the guard cell wall, Ψ_wall_, is brought under direct experimental control.

## Materials and Methods

### Plant propagation and epidermal peels

Broadbean (*Vicia faba* L.) var. Bunyards Exhibition were grown from seed on soil under a 9 h : 15 h, 22°C : 18°C, light : dark cycle with 150 μmol m^−2^ s^−1^ photosynthetically active radiation (PAR) and 60% RH. Epidermal peels of roughly 1–1.5 cm^2^ were prepared from young, fully expanded leaves as described previously (Blatt, 1987) and were mounted across 6‐mm‐diameter apertures punched out of precut, 2‐cm circles of TESA 64621 double‐sided plastic tape (Tesa, Hamburg, Germany). Once mounted, peels were immediately washed with distilled water before pretreating for 2 h under 150 μmol m^−2^ s^−1^ PAR while bathed in modified stomatal opening buffer (Eisenach *et al*., 2012) comprising 10 mM MES‐KOH, pH 6.1 with 5 mM KCl. For some experiments, pretreatments were carried out under defined pCO_2_ between 0 and 1000 μbar.

### Gas exchange analysis

**Fig. 2:**
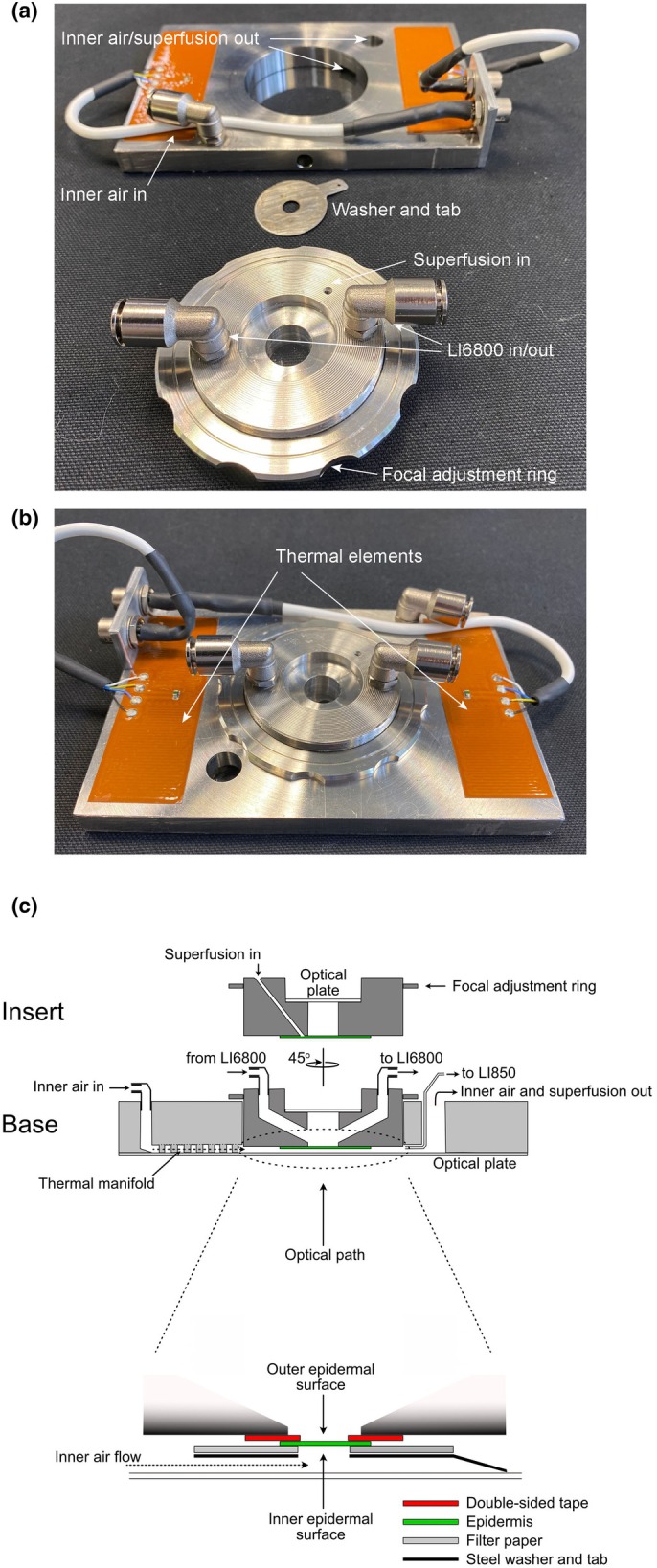
Bespoke chamber for analysing gas exchange across isolated epidermis. (a) Disassembled, the principal components comprise a base thermal block fixed on an optical plate (above), a washer and tab (centre), and a circular insert keyed to the block with a focal adjustment ring (below). The thermal block includes ports for air flow across the inner epidermal surface and for removing superfusing solution. The insert incorporates an optical plate above, two ports to enable air flow over the outer surface of the epidermis, a port for superfusion input, and is threaded for positioning of the focal adjustment ring. (b) Assembled, all ports and thermal elements are clearly visible. The washer (not visible) affixes to the lower surface of the insert and is held in place with two neodymium magnets mounted into the lower face of the insert. (c) Cross‐sectional schematic view of the assembled chamber showing the thermal block (Base, light grey) and insert (Insert, dark grey) with each of the ports labelled. The expanded view (below) shows the assembly with epidermis affixed to double‐sided tape, and sandwiched with filter paper for wetting held in place by the washer. The washer tab makes contact with the lower optical plate by the open port for inner air and superfusion outflow, the tab facilitating the latter.

Gas exchange measurements made use of the custom‐built chamber described in Fig. 2. For each experiment, a Tesa tape‐mounted peel was secured over the primary opening of the chamber, thereby dividing the chamber between two compartments. The upper compartment (Insert), 3 ml in volume, faced the outer surface of the peel and was connected to a LICOR 6800 gas exchange system fitted with a LI6800‐19 custom chamber attachment (LICOR, Lincoln, NE, USA). The lower compartment (Base), also 3 ml in volume, faced the inner surface of the peel and was formed within a 400‐g thermal block of stainless steel, sealed below against 0.4‐mm‐thick optical glass and above by the upper compartment insert. The lower compartment was connected to a LICOR 610 dewpoint generator, the air intake of which was connected to a mixing chamber fed from compressed air tanks to provide 20% O_2_ and 80% N_2_ with defined pCO_2_ between 0 and 1000 μbar. Thus, air flow through the lower chamber was controlled for both CO_2_ and water vapour content. Both upper and lower compartments were maintained at atmospheric pressure, and the outflow of the lower compartment was monitored for CO_2_ and water vapour content with a LICOR 850 gas analyser.

Once mounted, each peel was overlaid on the inner compartment side with a surrounding 2.5‐cm‐diameter disc of wetted filter paper (Whatman no. 1, Whatman, Cheshire, UK), prepunched with a 6‐mm aperture. The filter paper assembly was held in place by two neodymium magnets (60 g force, Eclipse, Sheffield, UK) and a 0.2‐mm‐thick steel washer with a matching, 6‐mm‐diameter aperture. The washer included a 1‐cm tag to draw excess solution away from the peel for aspiration and the stainless steel insert incorporated a 2‐mm‐diameter port for adding solution to the filter paper.

Fully assembled, the chamber was adjusted to position the epidermal peel 1.5–2 mm above the lower chamber window for microscopic image acquisition concurrent with gas exchange measurements. Stomata were observed using a Zeiss IM200 inverted microscope fitted for Nomarski differential interference contrast imaging with 10×/0.25NA and LD 20x/0.4NA objectives, and all experiments were carried out under continuous white light of 100 μmol m^−2^ s^−1^ from the halogen lamp of the microscope. Images were collected using an Amscope MU1803‐CK 18 MPixel CMOS camera (Amscope, Dorchester, UK) for later analysis of apertures.

Experiments were run at a temperature of 20°C, two degrees above ambient. Chamber design ensured upper and lower compartments were in thermal contact and provided for gas flow over a 50‐cm^3^ path within the stainless steel block enabling temperature to be regulated to ±0.05°C by a pair of thermal elements and TC300 controller (Thorlabs, Newton, MA, USA). All gas lines were insulated and thermally regulated to match the block temperature set for the chamber. Airflow sampled by the LI850 gas analyser was warmed by 1–2°C above that of the chamber flow to avoid condensation while still providing readings for absolute water content and allowed for monitoring values above 99% RH. Given the lack of stomatal responsiveness, even with very much lower RH, these values were rounded up for reporting.

### Stomatal apertures

Apertures were determined from images collected during each experiment as described previously using ImageJ (Rasband & Bright, 1995; Schindelin *et al*., 2012) after calibration. Typically, 15–30 stomata were visible in any one field of view, and three fields of view were carried through analysis so that each experiment followed both total *g*_s_ and the apertures of 45–90 stomata.

### Microtensiometry

Microtensiometers were constructed, by analogy with microcapillary pressure gauges (Tomos, 2000; Knoblauch *et al*., 2014), using fine capillary glass, hand‐drawn from 3‐mm Kimax glass tubing (Merck, Gillingham, UK) to give fine capillaries of 150–300 μm diameter. These were cut to lengths of 8–10 mm, and fire‐sealed at one end. The capillaries were filled with degassed, distilled water by submersion under low vacuum and vacuum release. Thereafter, the open end of each capillary was enclosed by dipping in melted 3% (w/v) agar (Merck) and allowing the agar plug to solidify before storing in distilled water.

Capillary microtensiometers retained a small bubble at the fire‐sealed end Supporting Information (Fig. S1). The bubble meniscus expanded along the length of the capillary when the water potential outside the agar plug was lowered, effectively reporting the external water potential balanced by a partial vacuum within the capillary. From the van't Hoff relation, then, the external water potential:

(Eqn 1) $$\Psi =-\Psi_{P}=\Psi_{\pi }=-i\mathrm{CRT}$$

where −Ψ_P_ is the potential associated with the partial vacuum, Ψ_π_ is the osmotic potential introduced by the solute, *i* is the solute dissociation factor, *C* the solute concentration, and R and T are the universal gas constant and temperature in ^o^K, respectively. The bubble retained in distilled water indicated the presence of a volume of air when Ψ = 0 and introduced an offset to Eqn (1). Nonetheless, readings of the meniscus were reproducible. Each microtensiometer was calibrated by superfusion with solutions of 100–800 mM mannitol and of 0.1–1 M KCl that gave defined Ψ_π_ with values between 0 and −7 MPa. Microtensiometers with response halftimes of 3 min or less were selected for use. Prepared in this way, each microtensiometer was reusable for 3–5 d when stored in distilled water between experiments.

Microtensiometers were necessarily held by the steel washer with the agar plug at the edge of the chamber aperture between the filter paper and epidermal peel. This arrangement ensured the capillaries were positioned across the chamber aperture to allow visual monitoring along with stomatal apertures. With the plug in contact with the epidermis and filter paper, the assembly reported bulk Ψ_wall_ of the epidermis and that of the filter paper, not specifically of the guard cells. Nonetheless, it was reasonable to expect little difference in these values so long as hydration flow was maintained. When hydration was suspended, Ψ_wall_ of the epidermis at the centre of the chamber aperture may have declined more rapidly than at its edge. However, any such differences must have decreased as the entire assembly (paper and epidermis) dried.

### OnGuard modelling and statistics

Quantitative modelling was conducted using OnGuard3e (Chen *et al*., 2012; Hills *et al*., 2012; Wang *et al*., 2017; Jezek *et al*., 2017, 2019, 2021; Nguyen *et al*., 2023). OnGuard3e models used the standard wild‐type (WT) parameter set for *Vicia*, scaled from the corresponding set for Arabidopsis, and were driven through a diurnal light : dark cycle as described previously (Chen *et al*., 2012; Hills *et al*., 2012; Wang *et al*., 2017; Jezek *et al*., 2019, 2021; Nguyen *et al*., 2023). All model outputs were derived from the diurnal cycle. Changes in atmospheric RH (*w*_atm_) and RH in the substomatal cavity (*w*_*i*_) were imposed on this cycle as indicated, the latter by adjusting the relative water feed (RWF) to the leaf or its fractional contribution to Ψ_wall_ according to the relation (Wang *et al*., 2017)

(Eqn 2) $$C_{\mathrm{iso}}={C_{\mathrm{iso}}}^{o}+{\left(1/v_{L}\right)}^{\cdot }\ln\left(w_{\mathrm{sat}}/w_{i}\right)/F_{M}$$

where *C*_iso_ substitutes for *C* in Eqn (1). Here, *C*_iso_^o^ is the dissolved solute concentration in the cell wall, *v*_L_ is the molar volume of water, and *F*_M_ is a coupling (attenuation) factor, normally of unity, that defines the distance between the guard cell and the point defining substomatal *w*_*i*_. Constant apoplastic solute contents were defined, and primary, energy‐dependent transport, sucrose and malic acid synthesis within the guard cell were coupled to light as before. Light input also contributed to the rate of carbon fixation by the leaf mesophyll and, hence, to the sink for CO_2_ within the leaf according to established relationships between light, CO_2_ and carbon assimilation (Jezek *et al*., 2021). All other model parameters were fixed. The properties of the individual transporters, metabolism and buffering reactions, and water flux and vapour exchange thus responded only to changes in model variables arising from the parameters encoded in the model. OnGuard3e and the relevant parameter set (Notes S1) are freely available for academic users for download from wwwplantscienceglasgow.org.

As OnGuard outputs are determined by the interactions of the ordinary differential equations that describe each of the underlying processes, statistical analysis of these outputs is meaningless. Where relevant, results are reported as mean ±SE of *n* independent experiments. Significance was determined by one‐way analysis of variance with *post hoc* analysis (Holm‐Sidek and Tukey) and is indicated at *P* < 0.05 unless otherwise stated.

## Results

Our approach was to measure gas exchange directly across the isolated epidermis of leaves mounted and sealed within a temperature‐controlled, double‐compartment chamber (Fig. 2) analogous to that used by Lange *et al*. (1971). For our measurements, one compartment of the chamber, facing the inner surface of the epidermis (hereafter the inside), was supplied with a constant stream of air of defined pCO_2_ (pC_*i*_) with *w*_*i*_ controlled by a LICOR 610 dewpoint generator. This first compartment included ports for superfusion to wet the epidermis and for outflow to remove excess solution, enabling control of cell wall hydration during experiments. Air passing through the outflow was also sampled using a LICOR 850 gas analyser to determine *w*_*i*_ and pC_*i*_ on the inside of the epidermis. The second compartment, facing the external surface of the epidermis (hereafter the outside), was connected to a LICOR 6800 infrared gas analyser with a custom chamber adapter. When assembled, the chamber allowed continuous visual monitoring of stomatal apertures concurrent with recordings of stomatal transpiration using a microscope‐mounted CMOS camera.

We used leaf epidermis of broadbean (*Vicia faba* L.) from which peels of 1 cm^2^ and more are easily obtained with stomata intact and functional (Blatt, 1987; Brearley *et al*., 1997), thus large enough to seal across the 6‐mm‐diameter aperture separating the chamber compartments. Initially we maintained the temperature at 20°C with a flow inside of 0.4 l min^−1^, roughly equivalent to 2 compartment volumes per second, holding *w*_*i*_ at 2.34 kPa (100% RH; *w*_*i*_ *≈ w*_sat_) and pC_*i*_ at 250 μbar. The epidermis was wetted with a minimal buffer solution of 0.5 mM Ca^2+^‐MES, pH 6.1 (=0.1 mM Ca^2+^) and 10 mM KCl to which further additions were made as required. The outside was held at 400 μbar pCO_2_ and 50 %RH (*w*_atm_ = 1.17 kPa). Stomatal conductance was calculated in the usual way, as follows:

(Eqn 3) $$g_{s}=E^{\cdot }P_{\mathrm{air}}/\left(w_{i}-w_{\mathrm{atm}}\right)=E^{\cdot }P_{\mathrm{air}}/\Delta w$$

where *E* is the rate of transpiration, *P*_air_ is the total air pressure, *w*_atm_ is the partial pressure of water vapour in the atmosphere, and *w*_*i*_ is the partial pressure of water vapour within the leaf in the substomatal cavity.

**Fig. 3:**
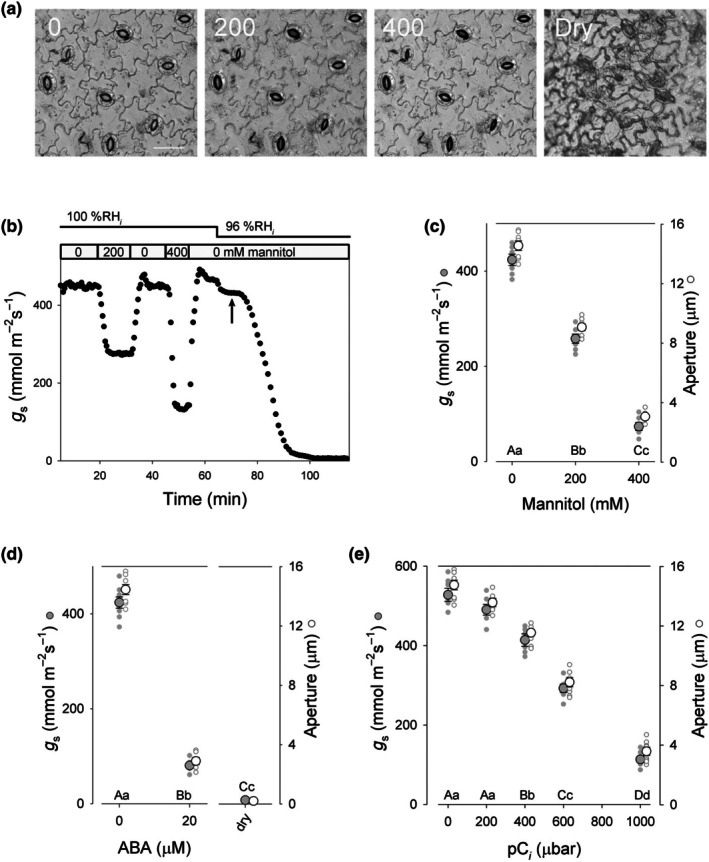
Aperture and conductance (*g*_s_) responses of *Vicia faba* epidermis to mannitol, abscisic acid (ABA) and internal CO_2_ partial pressure. (a) Images collected from one experiment while wetting with 0.5 mM Ca^2+^‐MES, pH 6.1 (=0.1 mM Ca^2+^) and 10 mM KCl alone and with additions of 200 and 400 mM mannitol, its washout, and drying of the epidermis. Images are labelled to indicate the mannitol concentration and the view on drying. Bar, 50 μm. (b) *g*_s_ recorded together with the images in (a) and calculated from Eqn (3) assuming *w*_*i*_ = *w*_sat_. The arrow marks the time point at which superfusion was stopped. (c, d) Summary of the data in (a, b) together with the results of seven additional experiments along with data from separate experiments before and after superfusing with 20 μM ABA. In each case, small symbols are individual experimental data and larger symbols are the corresponding means ±SE. (e) Summary of steady‐state apertures and *g*_s_ to the CO_2_ partial pressure inside (pC_*i*_) between 0 and 1000 μbar. Significant differences (c–e; *P* < 0.02) are indicated by lettering (upper case, *g*_s_; lower case, aperture).

Fig. 3a and b show an example of a recording and corresponding images collected from one experiment on wetting the epidermis with buffer without and with 200 and 400 mM mannitol, its washout and drying of the epidermis. These, and additional data, are summarised in Fig. 3c,d including experiments adding the water‐stress hormone abscisic acid (ABA) to trigger stomatal closure. As expected, increasing concentrations of mannitol reduced *g*_s_ and stomatal apertures, with substantial reductions in both evident with 200 and 400 mM mannitol. Adding 20 μM ABA similarly led to stomatal closure, with declines in *g*_s_ to values near 100 mmol m^−2^ s^−1^. Background *g*_s_ values near 20 mmol m^−2^ s^−1^ were recorded on complete and irreversible drying of the epidermis (Fig. 3a,b,d) and likely represent the residual water loss through the epidermal wall and cuticular layer. Increasing pC_*i*_ across the range from 0 to 1000 μbar (Fig. 3e) similarly reduced *g*_s_ and stomatal apertures. In each case, the dynamic ranges in aperture and *g*_s_ were displaced overall relative to measurements from the intact leaves (Gorton *et al*., 1993; McAusland *et al*., 2016; Santos *et al*., 2021). These differences may be ascribed to a loss in backpressure from the surrounding epidermal cells, the majority of which do not survive peeling (Allaway & Hsiao, 1973; Blatt, 1987), so that the stomata are able to achieve greater apertures than in the intact leaf. The responsiveness of the stomata therefore lent confidence that the measurements were physiologically meaningful.

**Fig. 4:**
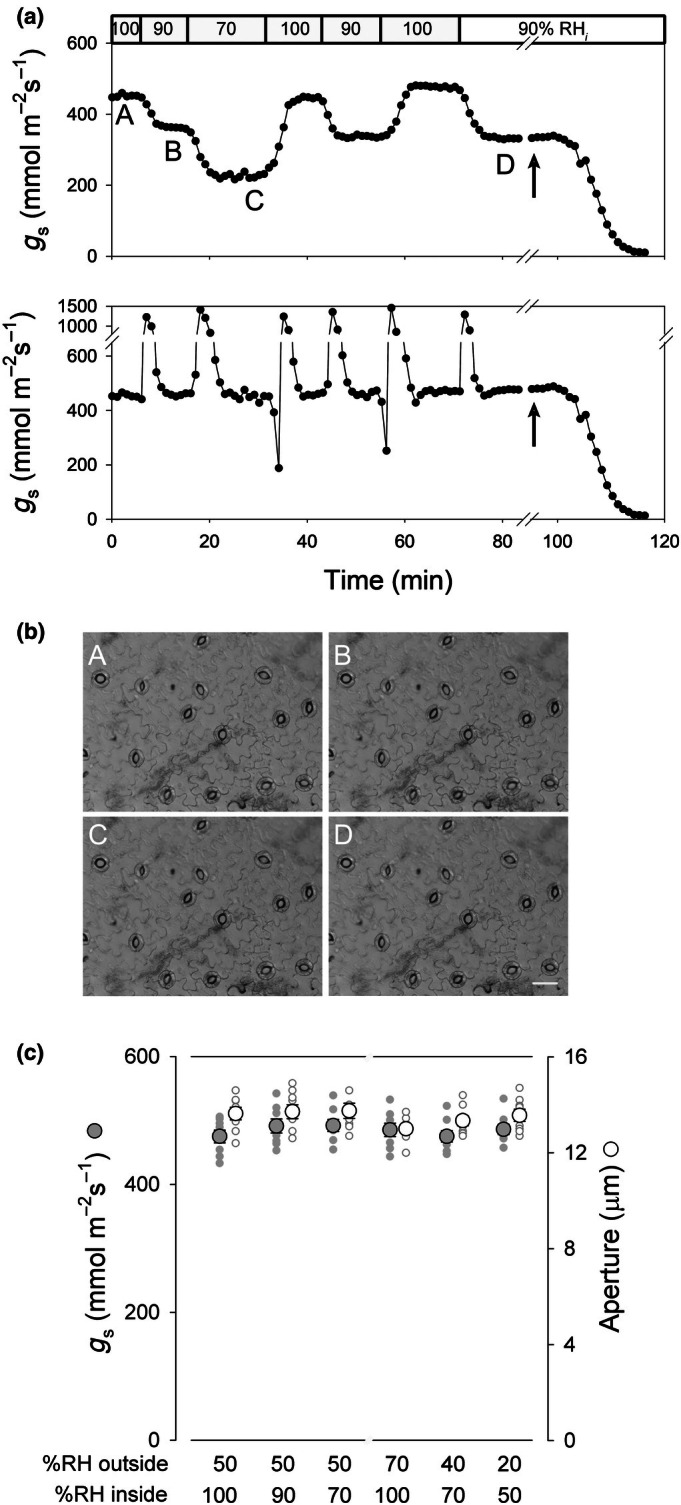
Aperture and conductance (*g*_s_) responses of *Vicia faba* epidermis to internal water vapour content. (a) *g*_s_ collected from one experiment with 50% relative humidity (RH) (*w*_atm_ = 1.17 kPa) outside and %RH inside (RH_*i*_) stepped between 100%, 90% and 70% (*w*_*i*_ = 2.34, 2.11 and 1.64 kPa). The epidermis was wetted with 0.5 mM Ca^2+^‐MES, pH 6.1 (=0.1 mM Ca^2+^) and 10 mM KCl. Arrows indicate when superfusing was stopped to allow drying of the epidermis. The partial pressure of CO_2_ was maintained at 250 μbar inside and 400 μbar outside. Values for *g*_s_ were calculated from Eqn (3) assuming *w*_*i*_ = *w*_sat_ inside (above) and using the measured values for *w*_*i*_ (below). Transients apparent with the measured values for *w*_*i*_ arise from the calculations and are not meaningful. (b) Images collected from the experiment in (a). Labels indicate the water vapour partial pressures and the view on drying. Bar, 50 μm. (c) Summary of steady‐state apertures and *g*_s_ as a function of *w*_*i*_ (left), including the data of (a), and as a function of *w*_*i*_ and *w*_atm_ with Δ*w* held constant at 0.70 kPa (right). Small symbols are individual experimental data (*n* = 8) and larger symbols are the corresponding mean ± SE. No significant differences were recovered in either set of experiments.

We noted that reducing *w*_*i*_ to 2.25 kPa (=96 %RH_*i*_) had little effect on *g*_s_ until epidermal wetting was stopped and the epidermis began to dry out (Fig. 3b). We therefore examined the effects of reducing *w*_*i*_ while maintaining 250 μbar pC_*i*_ and wetting the epidermis with 0.5 mM Ca^2+^‐MES and 10 mM KCl as before. As expected, *g*_s_ declined with experimentally controlled reductions in *w*_*i*_ (or %RH) when calculated from the transpiration rates assuming saturating *w*_*i*_ (=*w*_sat_) (Fig. 4a, above). However, correcting for the true *w*_*i*_ sampled from inside, and hence for the reduced diffusional driving force Δ*w*, gave results showing no appreciable change in *g*_s_ and aperture measurements (Fig. 4a below, and Fig. 4b,c) confirmed that the stomata remained open, even with 70 %RH_*i*_ (*w*_*i*_ = 1.64 kPa). Equivalent results were obtained when measurements were carried out after reducing the gaseous flow on the inside to 0.1 l min^−1^ and when the flow was increased to 1 l min^−1^ (Fig. S2), indicating that the measurements were not subject to differences in laminar gradients arising with gaseous flow across the inside of the epidermis. To separate the effects of changing Δ*w* from *w*_*i*_, we also reduced *w*_atm_ in parallel with *w*_*i*_ to maintain a constant Δ*w* of 0.70 kPa. Comparing steady‐state *g*_s_ values in this case (Fig. 4c) showed no significant differences, even when *w*_*i*_ was reduced to 1.17 kPa (50 %RH_*i*_; 20% RH_o_ and *w*_atm_ = 0.47 kPa). Indeed, so long as the epidermal cell wall space was wetted, we observed no significant effect of *w*_*i*_ on *g*_s_ or stomatal aperture.

To explore the consequences of reducing *w*_*i*_, we used the OnGuard3e platform (Blatt *et al*., 2022; Nguyen *et al*., 2023) to calculate *g*_s_ using both *w*_*i*_ = *w*_sat_ and *w*_*i*_ estimated from the diffusional resistances across the path from evaporation at the mesophyll cell surface to the atmosphere (Wang *et al*., 2017). The OnGuard platform assumes rapid osmotic equilibration between liquid water within the guard cell and in the surrounding cell wall. It incorporates the convention of Peak & Mott (2011) describing the path of resistance to water vapour diffusion between the evaporative surfaces of the leaf mesophyll and the atmosphere. In accord with this convention, it also defines a so‐called ‘p’ site (Peak & Mott, 2011) determining *w*_*i*_ within the substomatal cavity with which evaporative exchange occurs between water in the guard cell wall and water vapour within the substomatal cavity. Additionally, the platform includes a factor encompassing leaf hydration that is defined by the RWF. The RWF is expressed as the ratio of the evaporative surface within the leaf beneath each stoma divided by the area of the stomatal pore (Wang *et al*., 2017). In practice, values for RWF > 40 correspond to a water‐replete plant, while values for RWF < 10 correspond to a plant under water stress (Wang *et al*., 2017).

**Fig. 5:**
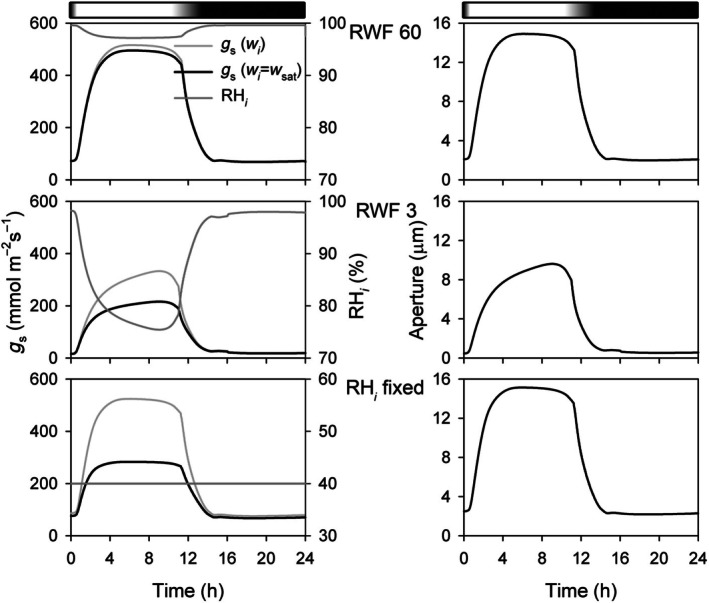
OnGuard simulations predict substantial declines in *w*_*i*_ and errors in *g*_s_ when vapour pressure differences are large and the plant is under water stress. OnGuard3e outputs for diurnal stomatal conductance (*g*_s_) and relative humidity (RH_*i*_) within the *Vicia* leaf airspace (left), and for stomatal aperture (right) in the well‐watered (relative water feed (RWF) 60, above) and water‐stressed (RWF 3, centre) leaf. Also shown are outputs for RWF 60 with RH_*i*_ clamped to 40 %RH (RH_*i*_ fixed). Diurnal light cycle with a graded transition between light and dark indicated above. Atmospheric relative humidity (RH_o_) set to 30% in each case. Simulations with RH_o_ of 70% gave outputs visually indistinguishable from those shown for RWF 60 with 70% RH_o_. The full set of OnGuard outputs are included in Supporting Information Fig. S3 and show a substantial reduction in transpiration (*E*), arising with the displaced conductance profile to water vapour within the leaf airspace and reduced diffusional gradient across the pore (Fig. 1). Note that stomatal aperture is unaffected with the clamp to 40 %RH_*i*_ in the water‐replete leaf, although Eqn (3) severely underestimates *g*_s_ with the assumption that *w*_*i*_ = *w*_sat_. The parameter set used for modelling is listed in Notes S1.

As expected, OnGuard outputs showed little difference between calculations either with small or large values for Δ*w* so long as the leaf was well‐hydrated (RWF = 60). However, the outputs showed a substantial underestimate of *g*_s_ using *w*_*i*_ = *w*_sat_ when Δ*w* was large and *w*_*i*_ was reduced by decreasing RWF (Figs 5, S2). In other words, OnGuard predicted a large reduction in *w*_*i*_ under water stress, with values equivalent to 80 %RH and below with RWF of 3–5. The immediate effect was to reduce transpiration through the stomata by virtue of the decline in diffusional driving force across the pore (Fig. S2). Most important, OnGuard simulations also predicted that with water in cell wall (RWF = 60), there should be little change in stomatal aperture, even when *w*_*i*_ is effectively clamped with *w*_*i*_ < <*w*_sat_ (Fig. 5). In other words, water feed through the epidermis was predicted to shield the guard cells by maintaining Ψ_wall_ ≈ Ψ_cyt_, even when *w*_*i*_ declined far below *w*_sat_.

The osmotic solute content of water in the cell wall affects the vapour pressure of air at equilibrium as well as stomatal aperture. The vapour pressure *w* at equilibrium over a solution of a unimolecular (nondissociating) solute is related by Raoult's law to the mole fraction *x*_w_ of water in solution in which it is dissolved as follows:

(Eqn 4a) $$w={w_{\mathrm{sat}}}^{\cdot }x_{w}$$

with

(Eqn 4b) $$x_{w}=n_{w}/\left(n_{w}+n_{i}\right)$$

where *n*_w_ and *n*_*i*_ are the moles of water (=18.2 g mol^−1^) and of the dissolved solute, respectively, per unit volume. Thus, a solution of 400 mM mannitol, sufficient to reduce *g*_s_ and stomatal apertures to roughly 25% of the values recorded with 0.5 mM Ca^2+^‐MES and 10 mM KCl (Fig. 3a–c), is equivalent at equilibrium to a decline of 0.8 %RH – so that *w*_*i*_ = 0.992·*w*_sat_. For *w*_*i*_ of 1.17 kPa (50 %RH_*i*_) at equilibrium, the same relation would imply an impossibly high concentration of 55 M mannitol (the solubility limit for mannitol is 0.98 M in water at 20°C). Yet no significant effect was seen in *g*_s_ or stomatal aperture when *w*_*i*_ was set to 1.17 kPa (50 %RH_*i*_). Clearly, then, both conditions cannot be met. Stomatal conductance cannot be responsive to a small decline in *w*_*i*_ implicit with a decrease in apoplastic water potential on solute additions, yet be wholly nonresponsive to much larger declines with *w*_*i*_ thought to be in equilibrium with water in the cell wall. The simplest explanation is that the two experimental manipulations do not have the same impact on the water potential of the cell wall, Ψ_wall_.

Within the living leaf, the cell wall space normally contains water fed from the xylem. Water evaporates from the liquid phase in the wall to the air space within the leaf. At any given time, the majority of the water held in the leaf is contained within the cellular volume, including in the vacuoles. Thus, it is reasonable to assume that the water potential in the cytosol, Ψ_cyt_, is close to the bulk water potential of the tissue as a whole. Current gas exchange models assume that the vapour phase of the intercellular air space is in equilibrium with the bulk leaf water potential, and hence with the cytosol (Buckley, 2005; Damour *et al*., 2010; Peak & Mott, 2011; Buckley & Mott, 2013). It follows that Ψ_wall_ and Ψ_cyt_ in the guard cell are near equilibrium. Equilibrium between Ψ_wall_ and Ψ_cyt_ is consistent with the effects on *g*_s_ and stomatal aperture of adding apoplastic solute (Wang *et al*., 2017) (cf. Fig. 3a–c) and, as noted previously, it is consistent with OnGuard model outputs. A near‐equilibrium of Ψ_wall_ and Ψ_cyt_ also accords with measurements using fluorescent reporters (Jain *et al*., 2021), although high evaporative flux may lead to a decline in Ψ_wall_ below Ψ_cyt_ of the mesophyll (Jain *et al*., 2025).

Wong *et al*. (2022) have suggested that Ψ_wall_ may decline substantially below Ψ_cyt_ of the mesophyll under water stress by as much as −7 MPa, in order to buffer against the much larger depressions in *w*_*i*_. If the same is true for the guard cells, then we might expect that increasing the solute content of the cell wall should sensitise the stomata to the additional effects of *w*_*i*_ and its consequent impact on Ψ_wall_. We asked, therefore, whether a responsiveness of *g*_s_ to reduced *w*_*i*_ could be uncovered when the cell wall space was perfused with mannitol as an osmoticum. For these experiments, *w*_*i*_ was reduced while maintaining 250 μbar pC_*i*_, this time with *w*_atm_ held at 0.70 kPa (30% RH_o_) and with the epidermis wetted by 0.5 mM Ca^2+^‐MES, 10 mM KCl without and with the addition of 200 mM mannitol. Again, transpiration declined with decreasing *w*_*i*_ (Fig. S4), but no significant changes in stomatal aperture were evident and *g*_s_ similarly was unaffected after correcting for Δ*w*, even with *w*_*i*_ of 1.64 kPa (70 %RH_*i*_).

**Fig. 6:**
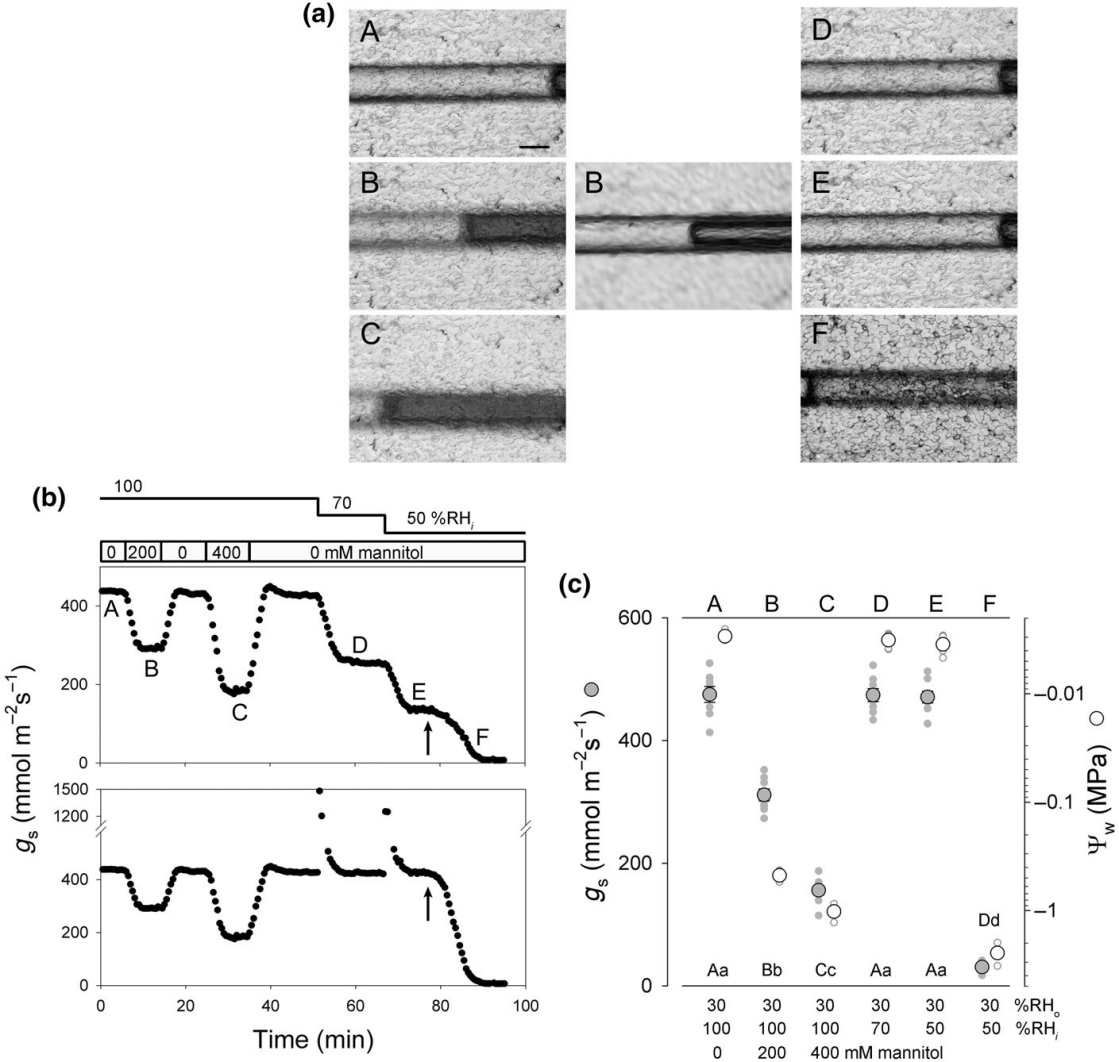
Stomatal conductance (*g*_s_) with microtensiometry of *Vicia faba* epidermal water potential (Ψ_w_) under challenge with mannitol and low internal water vapour content. (a) Images of an epidermal peel from one experiment with a microtensiometer before (A) and with mannitol additions (B, C), under reduced *w*_*i*_ (D, E), and after allowing the tissue to dry (F). Lettering cross‐relates to the time points in (b). Note the displacements of the meniscus in (B), (C) and (F). The central image (B) was collected with the focus adjusted on the microtensiometer. (b) *g*_s_ collected from the experiment of (a) with 30% relative humidity (RH) (*w*_atm_ = 0.70 kPa) outside and %RH inside (RH_*i*_) stepped between 100%, 70% and 50% (*w*_*i*_ = 2.34, 1.64 and 1.17 kPa). The epidermis was wetted with 0.5 mM Ca^2+^‐MES, pH 6.1 (=0.1 mM Ca^2+^) and 10 mM KCl. Arrows indicate when superfusion was stopped to allow drying of the epidermis. Letters indicate times when the images in (a) were collected. The partial pressure of CO_2_ was maintained at 250 μbar inside and 400 μbar outside. Values for *g*_s_ were calculated from Eqn (3) assuming *w*_*i*_ = *w*_sat_ inside (above) and using the measured values for *w*_*i*_ (below). Transients apparent with the measured values on steps in *w*_*i*_ arise from the calculations and are not meaningful. (c) Summary of steady‐state *g*_s_ as a function of *w*_*i*_ and Ψ_w_ (left), including the data of (b), with mannitol additions and steps in %RH_*i*_ (indicated below). Letters (A–F, above) cross‐relate to the image reference points in (a) and (b). Small symbols are individual experimental data (*n* > 6) and larger symbols are the corresponding means ± SE. Significant differences are indicated by lettering below (capitals, *g*_s_; lower case, Ψ_w_). Note the logarithmic scale for Ψ_w_.

Finally, we combined experiments controlling *w*_*i*_ while recording apertures and *g*_s_ together with concurrent measurements of the bulk Ψ_wall_. To assess Ψ_wall_, microtensiometers were constructed from fine glass capillaries closed at one end, filled with degassed, distilled water and sealed with a water‐permeable plug (Fig. S1). Each microtensiometer incorporated a small bubble that expanded reversibly with declines in Ψ_water_ in contact with the plug and was calibrated against known standards (see the Materials and Methods section). For the experiments, a microtensiometer was positioned with the bubble in the microscope field of view and the plug in contact with the epidermis and filter paper at the edge of the aperture separating the inner and outer chamber compartments. The measurements showed that additions of 200 and 400 mM mannitol yielded bulk Ψ_wall_ consistent with the change in osmotic potential, Ψ_π_. However, changes in *w*_*i*_, even when reduced to 1.17 kPa (50 %RH_*i*_), showed no resolvable declines in Ψ_wall_. Only when the tissue was allowed to dry was Ψ_wall_ seen to fall below −2 MPa, the stomata to close fully, and *g*_s_ to collapse to values near zero (Fig. 6). Thus, decreasing *w*_*i*_ did not reduce bulk Ψ_wall_ recorded by microtensiometry, nor were the stomata and *g*_s_ sensitised to *w*_*i*_ by reducing Ψ_wall_ with mannitol additions.

## Discussion

Caution is needed in extrapolating from the epidermal peel to stomatal function *in situ*. Not only are connections to the xylem and surrounding epidermal cells destroyed in peeling but also the underlying mesophyll is removed. These losses eliminate the normal hydraulic flow and greatly reduce the buffering volume of water normally held in the leaf tissues, as well as eliminating the impacts on pC_*i*_ of the photosynthetic carbon sink. Nonetheless, we can substitute for the hydraulic flux and the volume of mesophyll cell water by wetting and superfusion of an inert matrix in contact with the epidermis. Peeling offers some advantages in enabling direct experimental control of pC_*i*_ and *w*_*i*_. Introducing a continuous stream of gas to the inside of the epidermis also precludes the unstirred layers that might otherwise introduce uncertainties of the gaseous stationary fronts to which the stomatal guard cells are exposed.

Of course, these manipulations do not inform on *w*_*i*_ within the substomatal airspace of the intact leaf which, in general, is estimated indirectly from measurements of transpiration. However, they allow the experimenter to probe the range of *w*_*i*_ over which the stomata respond. Holloway‐Phillips *et al*. (2019) estimated that RH_*i*_ of *Vicia* would decline marginally to 97 %RH with high VPD. Thus, if *w*_*i*_ equilibrates with water in the wall space, we reasoned that reducing *w*_*i*_ much below this value should lead to rapid and complete stomatal closure. It was a surprise, therefore, to find that stomatal apertures were virtually insensitive to *w*_*i*_, even with values as low as 50 %RH inside, provided that the epidermal cell wall space was hydrated. If we assume that Ψ_cyt_ rapidly equilibrates with Ψ_wall_, the observations suggest that Ψ_wall_ was not greatly affected. The conclusion is consistent with fluorescent reporters (Jain *et al*., 2021) that have yielded values for Ψ_wall_ similar to bulk measurements from leaves (Johnson *et al*., 2009; Parent *et al*., 2009; Locke & Ort, 2015) with values between −0.5 and − 1 MPa, declining below −2 MPa under severe water stress. It accords also with our observations of stomatal closure on reducing Ψ_wall_ by adding mannitol and with microtensiometer measurements of Ψ_wall_ in the isolated epidermis. In other words, stomatal guard cells are sensitive to Ψ_wall_ and appear shielded from vapour phase water in the substomatal cavity by liquid water in the cell wall (Fig. 1).

Recent arguments have posited that Ψ_wall_ may fall substantially below that of the bulk leaf under water stress (Wong *et al*., 2022; Cernusak *et al*., 2024), proposing instead that distal positioning and regulation of aquaporins, both in the mesophyll and in the guard cells, prevents water efflux and cellular collapse, thereby allowing for Ψ_wall_ << Ψ_cyt_. Such arguments only transfer the problem within the cells, shifting the question of water flux to other pathways (Chaumont & Tyerman, 2014) including through ion channels (Woodbury & Hall, 1988; Homble & Very, 1992; Jensen *et al*., 2012). Even in the Arabidopsis *pip2;1* mutant that lacks the dominant guard cell aquaporin, stomata close in elevated pCO_2_, losing water at rates indistinguishable from that of WT plants, and closure is only slowed in ABA (Grondin *et al*., 2015). These observations indicate that water flux, at least across the guard cell plasma membrane, is not greatly constrained by loss of the aquaporin.

Underlying the estimates of a reduced Ψ_wall_ for the guard cell is the assumption that liquid water in the wall must always be in isothermal equilibrium with water vapour in the substomatal cavity. Our observations pose a challenge to this assumption: When *w*_*i*_ falls substantially below *w*_sat_, an equilibrium in water potential between the guard cell wall and substomatal cavity cannot be met while, at the same time, Ψ_wall_ is in equilibrium with Ψ_cyt_ of the turgid guard cell. Indeed, simulations with the OnGuard platform (Figs 5, S2) predict a steady‐state *w*_*i*_ that declines substantially below saturation even when Ψ_wall_ is in equilibrium with Ψ_cyt_ of guard cells around the open stoma.

Our experiments clearly show that Ψ_cyt_ and Ψ_wall_ equilibrate rapidly, both when *w*_*i*_ ≈ *w*_sat_ and when *w*_*i*_ << *w*_sat_, as evidenced by stomatal response to mannitol additions (cf. Figs 3, 6, S3). If Ψ_wall_ is in isothermal equilibrium with water vapour, then we can only surmise that the water vapour pressure must drop steeply from the point of evaporation to *w*_*i*_ in the substomatal cavity (Fig. 1). In other words, isothermal equilibrium in these circumstances must be constrained to a microscopic layer at the evaporative surfaces, with a substantial fraction of the total Δ*w* gradient arising immediately beyond the cell wall surface.

It may be that *Vicia* guard cells are partly shielded from the substomatal cavity by an inner‐facing cuticular layer over the cell wall. We are not aware of any ultrastructural studies that might speak to this question, but inner cuticular layers are known to occur in cotton (Wullschleger & Oosterhuis, 1989) and several genera within the Rosaceae and Asteraceae (Pesacreta & Hasenstein, 1999; Kumachova *et al*., 2022). Even if a cuticular layer does extend down the throat of the stomatal pore and over the inner surface of the guard cell wall, evaporation from the surrounding epidermal cell surface means that the guard cells will not be hydraulically isolated. In our hands, wetting with mannitol solutions around the chamber aperture yielded rapid changes in stomatal aperture and *g*_s_, and drying of both the epidermal and guard cells was evident within the chamber aperture when hydration was suspended (Figs 3, 4, 6, S4). In other words, with the defined gas flow across the inner epidermal surface, evaporation clearly draws water from the tissue, including the guard cells, whether the point of evaporation occurs at walls of the guard cells or neighbouring epidermal cells. Over the macroscopic scale of the substomatal cavity, then, evaporation from the cell wall may be better described as a flux in steady state driven by the gradient in potential from liquid water in the xylem and mesophyll cell wall space to water vapour in the substomatal cavity and across the stomatal pore (Fig. 1).

The interpretation of a steady state fits well with the scale of vapour flux. Under conditions of stable gas exchange, transpiration through the stomata, *E*, sets an upper limit on water evaporating from the cell wall surfaces within the leaf. The *V. faba* leaf is typically 300 μm thick with some 60% of the internal volume comprising airspace, giving a total leaf cell volume of *c*. 100 ml m^−2^. At a steady‐state *g*_s_ of 400 mmol m^−2^ s^−1^ and *E* of 5 mmol m^−2^ s^−1^ (Kaiser & Kappen, 1997; Mott, 2007; Grantz *et al*., 2018), transpiration loss voids an equivalent of the total volume of water in the leaf roughly every 20 min, in other words at a rate far in excess of what might be supported by changes in leaf cell turgor, even in the short term.

Given that this water must evaporate from the cell wall surface, the numbers are all the more stark. The evaporative rate from the cell wall, *E*_w_, is not known, but can be assumed to be an order of magnitude smaller than *E* through the stomatal pore to account for the greater evaporative surface behind the pore (Wang *et al*., 2017; Wong *et al*., 2022). The composition of the wall itself is complex but may be estimated to hold a volume of 50% water when hydrated (Sugiura *et al*., 2020; Evans, 2021). For a cell wall of 1 μm thickness (Sugiura *et al*., 2020), then, *E*_w_ implies the complete exchange of cell wall water roughly every 25 ms. These numbers indicate a large flux of water that is needed to maintain the steady state of cell wall hydration.

Finally, it is worth noting that Jain *et al*. (2025) observed a disequilibrium between Ψ_cyt_ and Ψ_wall_ in the mesophyll of maize leaves. They used a *slac1* mutant to ensure hyper‐open stomata and a very high evaporative flux, and they reported a recovery with Ψ_cyt_ ≈ Ψ_wall_, a loss of mesophyll turgidity and near‐collapse in transpiration when the hydraulic flux was suspended by cutting the leaf. Like our findings, these data underline the need to step beyond considerations of equilibria and address the steady‐state kinetics of liquid water flux and of evaporative diffusion in order to explain the observations.

In summary, we demonstrate a hydraulic exchange across the guard cell membrane, indicating that Ψ_wall_ ≈ Ψ_cyt_. Our results also show that this condition can hold even when *w*_*i*_ << *w*_sat_, in agreement with OnGuard predictions. The ability of cell wall water to support the open stoma, even in the face of low *w*_*i*_, accords with recent concepts of unsaturation within the substomatal cavity and a concomitant increase in the effective length for diffusion down the predominant water vapour gradient (Wong *et al*., 2022; Cernusak *et al*., 2024). However, it also implies that an equilibrium does not necessarily occur between the bulk liquid and gaseous phases of water within the leaf. The observations therefore challenge us to consider the kinetics of evaporative flux behind stomatal transpiration. Ultimately, it is this flux, away from equilibrium, that must determine stomatal gas exchange.

## Competing interests

None declared.

## Author contributions

MRB conceived the research and, with TL, designed the experiments. MRB and JM developed the chamber design that JM implemented. MRB developed the microtensiometer methods and carried out the experiments. MRB and AH carried out and analysed the OnGuard simulations. MRB wrote, and with TL and AH, edited the manuscript. All authors approved the manuscript.

## Disclaimer

The New Phytologist Foundation remains neutral with regard to jurisdictional claims in maps and in any institutional affiliations.

## Supporting information


**Fig. S1** A capillary glass microtensiometer.
**Fig. S2** Aperture and conductance (*g*_s_) responses of *Vicia faba* epidermis with differing inner gas flow rates.
**Fig. S3** OnGuard3e simulation of stomatal dynamics, transpiration and *w*_*i*_ using the *Vicia* parameter set.
**Fig. S4** Mannitol addition to the apoplast does not sensitise aperture and conductance (*g*_s_) to internal water vapour content.
**Notes S1** *Vicia* Guard Cell Model Parameters.
**Table S1** Tabulation of numerical data from Figs 3, 4 & 6.
Please note: Wiley is not responsible for the content or functionality of any Supporting Information supplied by the authors. Any queries (other than missing material) should be directed to the *New Phytologist* Central Office.

## Data Availability

All data for this publication are included in the figures and in tabular form in the Supplemental Information (Table S1). OnGuard3e is available together with the *Vicia* model parameters (Notes S1) from https://www.plantscienceglasgow.org. Further queries relating to the data may be made to Michael R. Blatt.
