# Supplementary material for: Cell wall water shields stomata against falling leaf airspace humidity
Source: New Phytol. 2026 Feb 8;250(2):861–72. doi: 10.1111/nph.70998 (PMC13001009; doi:10.1111/nph.70998)
Supplement: Supplementary file 1 — Fig. S1 A capillary glass microtensiometer. Fig. S2 Aperture and conductance (g s) responses of Vicia faba epidermis with differing inner gas flow rates. Fig. S3 OnGuard3e simulation of stomatal dynamics, transpiration and w i using the Vicia parameter set. Fig. S4 Mannitol addition to the apoplast does not sensitise aperture and conductance (g s) to internal water vapour content. Notes S1 Vicia Guard Cell Model Parameters. Table S1 Tabulation of numerical data from Figs 3, 4 & 6. Please note: Wiley is not responsible for the content or functionality of any Supporting Information supplied by the authors. Any queries (other than missing material) should be directed to the New Phytologist Central Office. [file NPH-250-861-s001.pdf]

New Phytologist Supporting Information
Article title: **Cell wall water shields stomata against falling leaf airspace humidity**
Authors: Michael R Blatt, Adrian Hills, Tracy Lawson, and Julie Magill
Article acceptance date: 14 January 2026

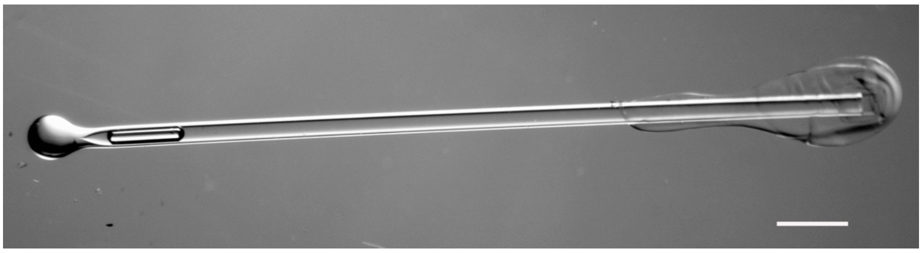

Supplemental Figure 1. **A capillary glass microtensiometer**
A microtensiometer comprising a fine capillary glass filament, fire-sealed at one end and plugged with 3% (w/v) agar at the other end. The capillary was filled with degassed, distilled water to leave a small bubble at the fire-sealed end of the capillary (see also Figure 6). Scale bar, 500 μm.

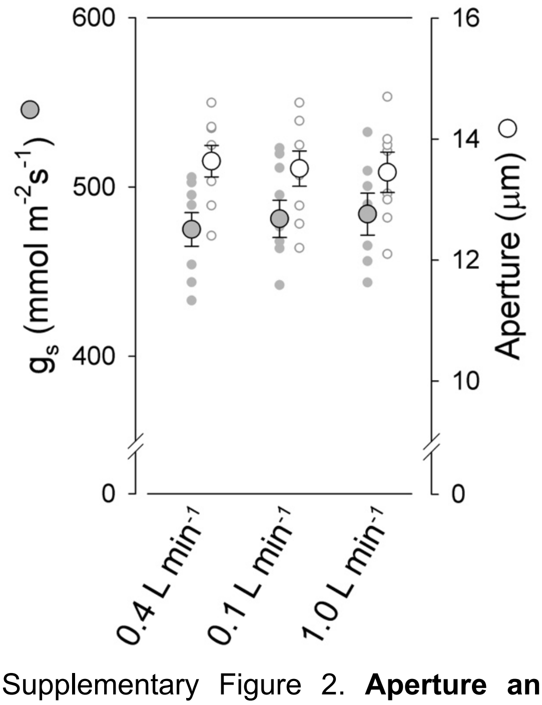

Supplementary Figure 2. **Aperture and conductance (g_s) responses of *Vicia faba* epidermis with differing inner gas flow rates.**
Data from eight experiments with epidermal tissue wetted with 0.5 mM Ca^{2+}-MES, pH 6.1 (=0.1 mM Ca^{2+}), and 10 mM KCl, as in Figure 3. Outside air held at 400 μbar CO_2 and 50% RH (w_{atm}=1.17 kPa); inside gas composition set to 250 μbar CO_2 and 70% RH_i (w_i=1.64 kPa). g_s calculated from Eqn [3] using the measured w_i. Small symbols are individual experimental data and larger symbols are the corresponding means ±SE. No significant differences were evident between inner gas flow rates of 0.1, 0.4 and 1.0 L min^{-1}.

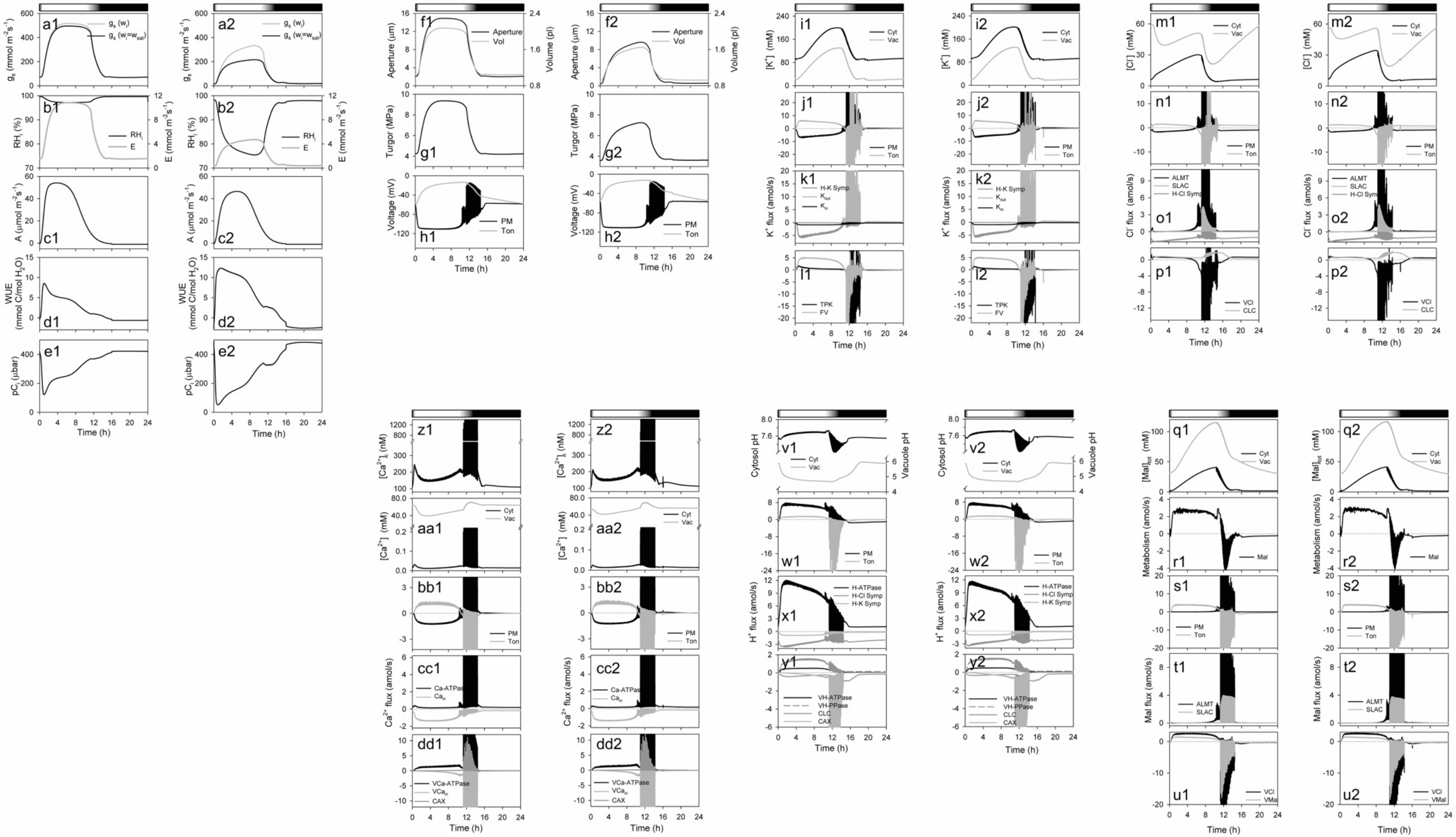

a1
a2
g_s (mmol m^{-2}s^{-1})
g_s (w_i)
g_s (w_i=w_{sat})
b1
b2
RH_i (%)
E (mmol m^{-2}s^{-1})
RH_i
E
c1
c2
A (µmol m^{-2}s^{-1})
d1
d2
WUE (mmol C/mol H_2O)
e1
e2
pC_i (µbar)
Time (h)
f1
f2
Aperture (µm)
Volume (pl)
Aperture
Vol
g1
g2
Turgor (MPa)
h1
h2
Voltage (mV)
PM
Ton
i1
i2
[K^+] (mM)
Cyt
Vac
j1
j2
k1
k2
K^+ flux (amol/s)
H-K Symp
K_{out}
K_{in}
l1
l2
TPK
FV
m1
m2
[Cl^-] (mM)
n1
n2
o1
o2
Cl^- flux (amol/s)
ALMT
SLAC
H-Cl Symp
p1
p2
VCl
CLC
z1
z2
[Ca^{2+}]$_i$ (nM)
aa1
aa2
[Ca^{2+}] (mM)
bb1
bb2
cc1
cc2
Ca^{2+} flux (amol/s)
Ca-ATPase
Ca_{in}
dd1
dd2
VCa-ATPase
VCa_{in}
CAX
v1
v2
Cytosol pH
Vacuole pH
w1
w2
x1
x2
y1
y2
H^+ flux (amol/s)
H-ATPase
H-Cl Symp
H-K Symp
VH-ATPase
VH-PPase
CLC
CAX
q1
q2
[Mal]$_{tot}$ (mM)
r1
r2
Metabolism (amol/s)
Mal
s1
s2
t1
t2
Mal flux (amol/s)
ALMT
SLAC
u1
u2
VCl
VMal

Supplementary Figure 3. **OnGuard3e simulation of stomatal dynamics, transpiration and w_i using the *Vicia* parameter set**

Parameters scaled from the corresponding standard Arabidopsis wild-type parameter set (Jezek *et al.*, 2021; Nguyen *et al.*, 2023). Bars (above) indicate the diurnal cycle. Simulations were run with 30 $\%RH_o$. Panels a1-dd1 are outputs with RWF 60 corresponding to the well-watered plant; panels a2-dd2 are outputs with RWF 5, corresponding to a water-stressed plant. Fluxes are indicated relative to the cytosolic compartment so that positive flux is movement out of the cytosol. Full descriptions of the transporters and their physiological characteristics will be found in Hills et al (2012) and Jezek and Blatt (2017). Clockwise from top left, outputs are grouped as:

(a) Stomatal conductance (g_s) calculated using w_i and w_i=w_{sat}; (b) relative humidity within the leaf ($\%RH_i$) and transpiration (*E*); (c) assimilation (*A*); (d) water use efficiency (WUE); and (e) CO_2 partial pressure within the leaf airspace (pC_i);

(f) stomatal aperture and guard cell volume; (g) guard cell turgor pressure; and (h) plasma membrane and tonoplast voltage;

(i) guard cell [K^+] in the cytosol (Cyt) and vacuole (Vac); (j) total K^+ flux across the plasma membrane (PM) and tonoplast (Ton); (k) plasma membrane flux through H^+-K^+ symport (H-K Symp), GORK (K_{out}) and KAT (K_{in}) channels; and (l) tonoplast K^+ flux through TPK- and FV-type channels;

(m) guard cell [Cl^-] in the cytoplasm (Cyt) and vacuole (Vac); (n) total Cl^- flux across the plasma membrane (PM) and tonoplast (Ton); (o) plasma membrane flux through H^+-Cl^- symport (H-Cl Symp), R-type (ALMT) and S-type (SLAC) channels; and (p) tonoplast Cl^- flux through H^+-Cl^- symport (CLC) and VCL-type channels;

(q) total guard cell [Mal] in the cytoplasm (Cyt) and vacuole (Vac); (r) Mal metabolism (synthesis, positive; breakdown, negative); (s) total Mal flux across the plasma membrane (PM) and tonoplast (Ton); (t) plasma membrane flux through R-type (ALMT) and S-type (SLAC) channels; and (u) tonoplast Mal flux through and VCL-type and VMal channels;

(v) guard cell pH in the cytoplasm (Cyt) and vacuole (Vac); (w) total H^+ flux across the plasma membrane (PM) and tonoplast (Ton); (x) plasma membrane H^+ flux through H^+-Cl^- symport (H-Cl Symp), H^+-K^+ symport (H-K Symp), and H^+-ATPases (H-ATPase); and (y) tonoplast H^+ flux through H^+-Cl^- symport (CLC), H^+-Ca^{2+} antiport (CAX), VH^+-ATPases (VH-ATPase) and VH^+-PPases (VH-PPase);

(z) guard cell cytosolic-free [Ca^{2+}] ([Ca^{2+}]$_i$); (aa) total [Ca^{2+}] in the cytoplasm (Cyt) and vacuole (Vac); (bb) total Ca^{2+} flux across the plasma membrane (PM) and tonoplast (Ton); (cc) plasma membrane flux through Ca^{2+}-ATPases (Ca-ATPase) and Ca^{2+} channels (Ca_{in}); and (dd) tonoplast Ca^{2+} flux through VCa^{2+}-ATPases (VCa-ATPase), Ca^{2+} channels (VCa_{in}), and H^+-Ca^{2+} antiport (CAX).

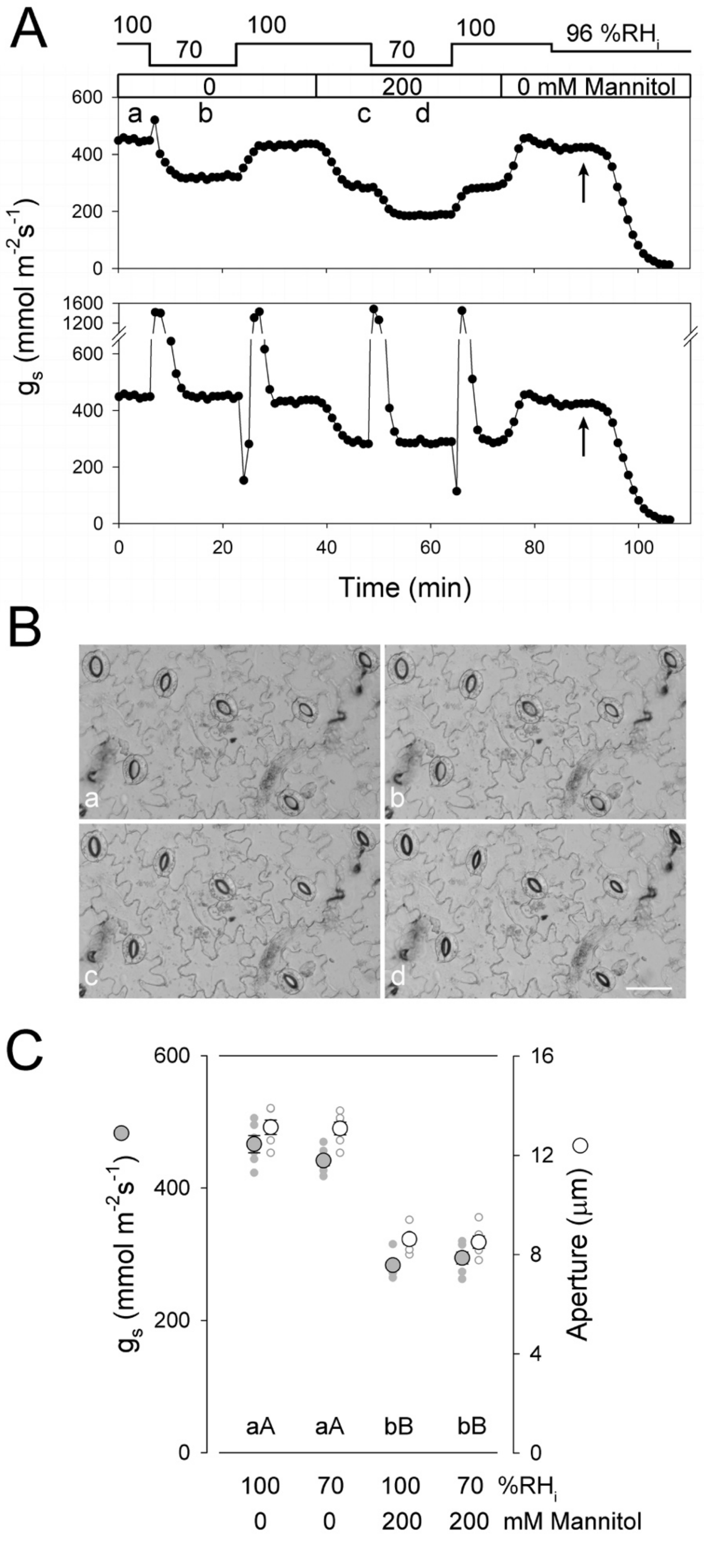

Supplemental Figure 4. **Mannitol addition to the apoplast does not sensitise aperture and conductance (*g_s*) to internal water vapour content**

(A) g_s collected from one experiment with 30% RH (w_{atm}=0.70 kPa) outside and %RH inside (RH_i) stepped between 100 and 70% (w_i=2.34 and 1.64 kPa). The epidermis was wetted with 0.5 mM Ca^{2+}-MES, pH 6.1 (=0.1 mM Ca^{2+}), and 10 mM KCl without and with 200 mM Mannitol. Arrow indicates when superfusing was stopped to allow drying of the epidermis. The partial pressure of CO_2 was maintaining at 250 μbar inside and 400 μbar outside. Values for g_s were calculated from Eqn [3] assuming w_i=w_{sat} inside (*above*) and using the measured values for w_i (*below*). Letters refer to the time of image collection (B).
(B) Images collected from the experiment in (A) cross-referenced by letter to the time of collection. Scale bar, 50 μm.
(C) Summary of steady-state apertures and g_s as a function of w_i (*left*), including the data of (A), and as a function of w_i and w_{atm} with Δw held constant at 0.70 kPa (*right*). Small symbols are individual experimental data (n=6) and larger symbols are the corresponding means ±SE. Letters (lower case, g_s; upper case, aperture) indicate significant differences at p<0.01. No significant differences were recovered with the change in water vapour content inside, either without or with mannitol.

Notes S1 Vicia Guard Cell Model Parameters
[Parameters used for RWF 60 and in **BOLD** used for RWF 3]

Total cell volume = 1.04758 pL; vacuolar fraction = 90.2939%

Stomatal Aperture Parameters:
SA:P 'm'= 0.4 atm/µm; SA:P 'n'= 3.4 atm; SA:V 'R'= 0.08 pL/µm SA:V 'S'= 0.88 pL
Pore Length: 18 µm; Pore Depth: 25 µm

Cytosolic Protein Buffering: [Pr] = 0.163816 mM, pIso = 6.8, ap = -71
Cytosolic Calcium Buffering: [Bu] = 0.362169 mM, K = 3e-06 M # Ca Sites = 10

Temperature: Leaf: 25∞C; Air: 25∞C
Current 'time' in model = 00:00:00.00

*** Compartmental Solutions (/mM)
==================================

	Apoplast	Cytosol	Vacuole
pH	6.500000	7.544131	5.889644
K	10.00000	103.8514	10.92188
Ca	1.000000	0.013888	63.93275
Cl	12.00000	6.143322	58.14943
Suc	0.010000	0.221584	0.002176
MH2	3.23e-07	1.12e-07	0.025899
MH	7.94e-06	0.000030	0.155931
M	0.009992	0.424742	48.14761
HCO3	0.020134	0.222875	0.004938
CO2	0.014308	0.014308	0.014308
Malates	0.010000	0.424772	48.32944

*** Plasma Membrane Transporters
================================

PM K-in Channel [8000 units] (Inward-Rectifying GHK Channel)

```
#'K'  G/Gmax = 9 pOhms
      2-State Voltage Gate: VΩ = -185 mV,
      Zg = +1.8
Light-Sensitive: NO!
Ligand-Gates:
  Ca-inhibited (cytosol): Kd= 3.3e-07, Hill= 4;
  H-activated (cytosol): Kd= 6e-08, Hill= 2;
  H-activated (apoplast): Kd= 1e-07, Hill= 1;
```

PM K-out Channel [600 units] (Outward-Rectifying GHK Channel)

```
#'K'  G/Gmax = 20 pOhms
      VΩ = +1 ◊ F/RT ◊ ln([K]apo/10mM)
      Zg = +2
Light-Sensitive: NO!
Ligand-Gates:
  H-inhibited (cytosol): Kd= 3e-08, Hill= 2;
  HCO3-activated (cytosol): Kd= 0.0003, Hill= 2;
```

```
R-Type Anion Channel [2400 units] (Outward-Rectifying GHK Channel)
--------------------
#'Cl' G/Gmax = 3.4 pOhms
#'M'  G/Gmax = 2 pOhms
      VΩ = +1 ◊ F/RT ◊ ln(1e-06mM/[H]cyt)
      Zg = -2
Light-Sensitive: NO!
Ligand-Gates:
  Ca-activated (cytosol): Kd= 6e-07, Hill= 4;
  H-activated (cytosol): Kd= 3e-08, Hill= 3;
  HCO3-activated (cytosol): Kd= 0.0003, Hill= 3;


V-Gated Ca-IN [24 units] (Inward-Rectifying GHK Channel)
-------------
#'Ca' G/Gmax = 12 pOhms
      VΩ = +0.5 ◊ F/RT ◊ ln(2e-09mM/[Ca]cyt)
      Zg = +1
Light-Sensitive: NO!
Ligand-Gates:
  Ca-inhibited (cytosol): Kd= 5e-07, Hill= 5;


Anion VIC [600 units] (Outward-Rectifying Ohmic Channel)
---------
#'Cl' G/Gmax = 0.15 pOhms
#'M'  G/Gmax = 0.07 pOhms
      Voltage-Independent
Light-Sensitive: NO!
Ligand-Gates:
  Ca-activated (cytosol): Kd= 6e-07, Hill= 4;
  H-activated (cytosol): Kd= 4e-08, Hill= 2;
  HCO3-activated (cytosol): Kd= 0.0003, Hill= 1;


H-ATPase [400000 units] (4-State 'Slayman' Pump)
--------
#'H'  Stoichiometry = +1; binds at 4->1 (in) and 3->2 (ex);
      K12 = 2000, K23 = 50000,     K34 = 500,  K41 = 2e+09,
      K21 = 100,  K32 = 1e+08,     K43 = 10,   K14 = 200;
Light-Sensitive: Yes: LΩ = 50 µEinsteins, Fmin = 5%
Ligand-Gates:
  Ca-inhibited (cytosol): Kd= 2.5e-07, Hill= 3;


H:Cl Symport [100000 units] (4-State Carrier)
------------
#'H'  Stoichiometry = +2; binds at 4->1 (in) and 3->2 (ex);
#'Cl' Stoichiometry = +1; binds at 4->1 (in) and 4->3 (ex);
      K12 = 1000, K23 = 100,  K34 = 50000,     K41 = 1e+21,
      K21 = 50,   K32 = 1e+21,     K43 = 100000,    K14 = 100;
Light-Sensitive: NO!
Ligand-Gates:
 <none>
```

```
H:K Symport [32000 units] (4-State Carrier)
-----------
#'H'  Stoichiometry = +1; binds at 4->1 (in) and 3->2 (ex);
#'K'  Stoichiometry = +1; binds at 4->1 (in) and 4->3 (ex);
      K12 = 2,    K23 = 10000,     K34 = 100000,    K41 = 1e+14,
      K21 = 0.4,  K32 = 1e+12,     K43 = 1e+10,     K14 = 50;
Light-Sensitive: NO!
Ligand-Gates:
 <none>


Ca-ATPase [60000 units] (4-State 'Slayman' Pump)
---------
#'Ca' Stoichiometry = +1; binds at 4->1 (in) and 3->2 (ex);
      K12 = 2000, K23 = 10000,     K34 = 500,  K41 = 1e+15,
      K21 = 2,    K32 = 1e+07,     K43 = 500,  K14 = 1000;
Light-Sensitive: Yes: LΩ = 50 µEinsteins, Fmin = 50%
Ligand-Gates:
  Ca-activated (cytosol): Kd= 5e-07, Hill= 2;
  HCO3-inhibited (cytosol): Kd= 0.0003, Hill= 4;


HMal symp [40000 units] (Concentration-Driven SYMPORT)
---------
#'H'  (Stoichiometry = -3)
#'M'  (Stoichiometry = -1)
      Fmax = 1e+20
Light-Sensitive: NO!
Ligand-Gates:
 <none>


K leak [2 units] (Inward-Rectifying GHK Channel)
------
#'K'  G/Gmax = 1 pOhms
      Voltage-Independent
Light-Sensitive: NO!
Ligand-Gates:
 <none>


*** Tonoplast Transporters
==========================

TPK1 [600 units] (Inward-Rectifying Ohmic Channel)
----
#'K'  G/Gmax = 90 pOhms
      Voltage-Independent
Light-Sensitive: NO!
Ligand-Gates:
  Ca-activated (cytosol): Kd= 3e-06, Hill= 1;
  H-activated (cytosol): Kd= 3e-08, Hill= 3;


TPC1 [100 units] (Outward-Rectifying GHK Channel)
----
#'Ca' G/Gmax = 27 pOhms
#'K'  G/Gmax = 14 pOhms
```

```
      VΩ = +1 ◊ F/RT ◊ ln([Ca]vac/2mM)
      Zg = +2
Light-Sensitive: NO!
Ligand-Gates:
  Ca-activated (cytosol): Kd= 3e-05, Hill= 1;
  H-activated (vacuole): Kd= 1e-06, Hill= 1;


FV K Channel [3200 units] (Inward-Rectifying GHK Channel)
------------
#'K'  G/Gmax = 6 pOhms
      2-State Voltage Gate: VΩ = -30 mV,
      Zg = +1
Light-Sensitive: NO!
Ligand-Gates:
  Ca-inhibited (cytosol): Kd= 2e-07, Hill= 1;
  H-inhibited (cytosol): Kd= 4e-07, Hill= 1;


VCL [600 units] (Inward-Rectifying GHK Channel)
---
#'Cl' G/Gmax = 40 pOhms
#'M'  G/Gmax = 10 pOhms
      VΩ = +1 ◊ F/RT ◊ ln([H]vac/0.005mM)
      Zg = -1
Light-Sensitive: NO!
Ligand-Gates:
  Ca-activated (cytosol): Kd= 1e-06, Hill= 1;


Vacuole H-ATPase [400000 units] (4-State 'Slayman' Pump)
----------------
#'H'  Stoichiometry = +2; binds at 4->1 (in) and 3->2 (ex);
      K12 = 100,  K23 = 1000, K34 = 0.5,  K41 = 1e+18,
      K21 = 10,   K32 = 1e+08,     K43 = 5,    K14 = 10000;
Light-Sensitive: Yes: LΩ = 50 μEinsteins, Fmin = 10%
Ligand-Gates:
 <none>


Vacuole H-PPase [1200000 units] (4-State 'Slayman' Pump)
---------------
#'H'  Stoichiometry = +1; binds at 4->1 (in) and 3->2 (ex);
      K12 = 1000, K23 = 1000, K34 = 1e+11,     K41 = 3e+09,
      K21 = 100,  K32 = 5e+09,     K43 = 1e+07,     K14 = 10000;
Light-Sensitive: Yes: LΩ = 50 μEinsteins, Fmin = 10%
Ligand-Gates:
  Ca-inhibited (cytosol): Kd= 1e-07, Hill= 1;
  K-activated (cytosol): Kd= 0.05, Hill= 1;


Vacuole Ca-ATPase [1400000 units] (4-State 'Slayman' Pump)
--------------------
#'Ca' Stoichiometry = +1; binds at 4->1 (in) and 3->2 (ex);
      K12 = 3000, K23 = 1000, K34 = 1000, K41 = 1e+09,
      K21 = 0.3,  K32 = 10000,     K43 = 10,   K14 = 10000;
Light-Sensitive: Yes: LΩ = 50 μEinsteins, Fmin = 50%
Ligand-Gates:
```

```
  Ca-activated (cytosol): Kd= 3.5e-07, Hill= 3;
  HCO3-inhibited (cytosol): Kd= 0.0003, Hill= 4;
  Ca-inhibited (vacuole): Kd= 0.04, Hill= 4;


Vac.CLC [120000 units] (4-State Carrier)
-------
#'H'  Stoichiometry = +1; binds at 4->1 (in) and 3->2 (ex);
#'Cl' Stoichiometry = -2; binds at 1->4 (in) and 2->3 (ex);
      K12 = 1000, K23 = 1e+09,     K34 = 100,  K41 = 1e+10,
      K21 = 1000, K32 = 1e+09,     K43 = 10,   K14 = 1e+11;
Light-Sensitive: NO!
Ligand-Gates:
  H-inhibited (cytosol): Kd= 5e-08, Hill= 2;


Tonoplast VCa [8 units] (Outward-Rectifying GHK Channel)
-------------
#'Ca' G/Gmax = 10 pOhms
      VΩ = +1 ◊ F/RT ◊ ln(10mM/[Ca]vac)
         + +0.5 ◊ F/RT ◊ ln(0.5mM/[HCO3]cyt)
      Zg = +4
Light-Sensitive: NO!
Ligand-Gates:
  Ca-activated (cytosol): Kd= 5e-07, Hill= 4;
T-deactivation: switch= [Ca]cyt; Threshold= 0.001mM, TΩ= 100000ms,
reset= 5%/0.0005mM


CAX [100000 units] (Concentration-Driven ANTIPORT)
---
#'H'  (Stoichiometry = -3)
#'Ca' (Stoichiometry = +1)
      Fmax = 1e+22
Light-Sensitive: NO!
Ligand-Gates:
  Ca-activated (cytosol): Kd= 3e-06, Hill= 1;
  H-inhibited (cytosol): Kd= 4e-08, Hill= 3;


ALMT-Mal [600 units] (Inward-Rectifying GHK Channel)
--------
#'M'  G/Gmax = 6 pOhms
      2-State Voltage Gate: VΩ = +0 mV,
      Zg = -2
Light-Sensitive: NO!
Ligand-Gates:
  H-inhibited (cytosol): Kd= 8.5e-08, Hill= 2;
  Ca-activated (cytosol): Kd= 1e-06, Hill= 1;


NHX [20000 units] (Concentration-Driven ANTIPORT)
---
#'H'  (Stoichiometry = -1)
#'K'  (Stoichiometry = +1)
      Fmax = 100000
Light-Sensitive: NO!
Ligand-Gates:
```

<none>

*** METABOLISM
==============

Total Malate (apo/cyt/vac) = 0.01 0.424772 48.3294 mM
Total Sucrose (apo/cyt/vac) = 0.01 0.221584 0.00217574 mM

Photosynthesis:
Suc s-max = 20 fmol/h, LΩ = 50 µE
Mal s-max = 0 fmol/h, LΩ = 50 µE
Light Type: Total

Sucrose Sink:
R-max = 10 fmol/h, KΩ = 1 mM

Suc <-> Mal Conversion:
R-max = 10 fmol/h, KΩ(S) = 0.1 mM, KΩ(M) = 10 mM
Mid-point pH = 7.7, pH gradient = +100

'Q10' Temperature Coefficient: 2

*** PHOTOSYNTHESIS & WUE PARAMETERS
===================================

Stomata/mm≤ = 60
Stomatal Length/µm = 18; Depth = 25
Subepidermal Depth/µm: = 300; Empty Space = 60%

CO2 Assimilation, A = AL ◊ AC - Rd [Where AL = {fL + Amax-v[(fL + Amax)≤-4T.fL.Amax]}/2T and AC = 1/[1 + Kc/(Cin-Cc)])]

Amax (µmol/m≤/s) = 80
f = 0.15; T = 0.9
Kc (/ppm) = 80; Cc (/ppm) = 8; Rd (µmol/m≤/s) = 1

System used for Ciso = Mott (p-site)
Wet Surface Area Coefficient (RWF) = 60 **[3]**
Mott Attenuator (Divisor) = 3

*** Constraint Relaxation & Recovery
====================================

Use CRR? Yes
Solutes to include: K
Epidermal Cell Capacity (Amax, /fmol) = 28
Adjacent Wall Capacity (Smax, /fmol) = 2
Max Recovery Rate (/fmol/sec) = 0.006; (Order = 1)
Current Contents = 100.0%
Apply Turgor-Sensitivity? Yes
Attenuator Mid-Point (atm) = 9; Attenuator Gradient (/atm) = 3

Tabulation of numerical data from Figures 3,4 and 6

Figure 3C	[Mann]	gs	±SE	aperture	±SE
	0	423.8867	12.0423	14.55	0.3174
	200	258.1132	9.6589	9.0688	0.2332
	400	103.441	8.4983	3.56	0.1636

Figure 3D	[ABA]	gs	±SE	aperture	±SE
	0	423.8867	12.0423	13.55	0.3174
	20	80.441	5.6536	2.7167	0.2242
	dry	8.0882	1.134	0.2	0.01

Figure 3E	pCi	gs	±SE	aperture	±SE
	0	527.7725	16.4104	14.75	0.3174
	200	490.1597	13.602	13.575	0.2711
	400	413.8867	15.655	11.55	0.2129
	600	292.4639	10.5144	8.225	0.3034
	1000	113.4526	8.0927	3.5848	0.2724

Figure 4C	50	50	50	70	40	20	%RH outside
	100	90	70	100	70	50	%RH inside
gs	474.8975	491.1163	491.9097	485.1367	474.9639	485.9526	
±SE	9.9139	11.0131	9.3607	10.3926	8.8986	8.1715	
aperture	13.635	13.7123	13.75	12.9975	13.3375	13.5598	
±SE	0.2604	0.2901	0.3133	0.1856	0.2129	0.2558	

Figure 6C	30	30	30	30	30	30	%RH outside
	100	100	100	70	50	50	%RH inside
	0	200	400			dry	
gs	474.8975	311.1163	155.6597	473.8867	470.7139	30.621	
±SE	12.8279	9.843	7.6492	10.4843	9.4377	3.1031	
Yw	2.94E-03	4.75E-01	1.023	3.19E-03	3.50E-03	2.4675	
±SE	7.18E-05	0.0133	0.0437	1.49E-04	2.11E-04	0.1339	

References

Hills A, Chen ZH, Amtmann A, Blatt MR, Lew VL. 2012. OnGuard, a Computational Platform for Quantitative Kinetic Modeling of Guard Cell Physiology. Plant Physiol 159(3): 1026-1042.

Jezek M, Blatt MR. 2017. The Membrane Transport System of the Guard Cell and Its Integration for Stomatal Dynamics. Plant Physiol 174(2): 487-519.

Jezek M, Silva-Alvim F, Hills A, Donald N, Ishka MR, Shadbolt J, He B, Lawson T, Harper JF, Wang Y, et al. 2021. Guard cell endomembrane Ca2+-ATPases underpin a 'carbon memory' of photosynthetic assimilation that impacts on water use efficiency. Nature Plants 7: 1301-1307.

Nguyen TH, Silva-Alvim FAL, Hills A, Blatt MR. 2023. OnGuard3e: A predictive, ecophysiology-ready tool for gas exchange and photosynthesis research. Plant Cell And Environment 46(11): 3644-3658.
